# Supplementary material for: Targeting mTORC2 in lung squamous cell carcinoma improves anti-tumor immunity through the PSGL-1-VISTA axis
Source: Cancer Gene Ther. 2025 Jul 10;32(8):899–910. doi: 10.1038/s41417-025-00934-4 (PMC12353870; doi:10.1038/s41417-025-00934-4)
Supplement: Supplementary file 1 — Supplementary Information [file 41417_2025_934_MOESM1_ESM.pdf]

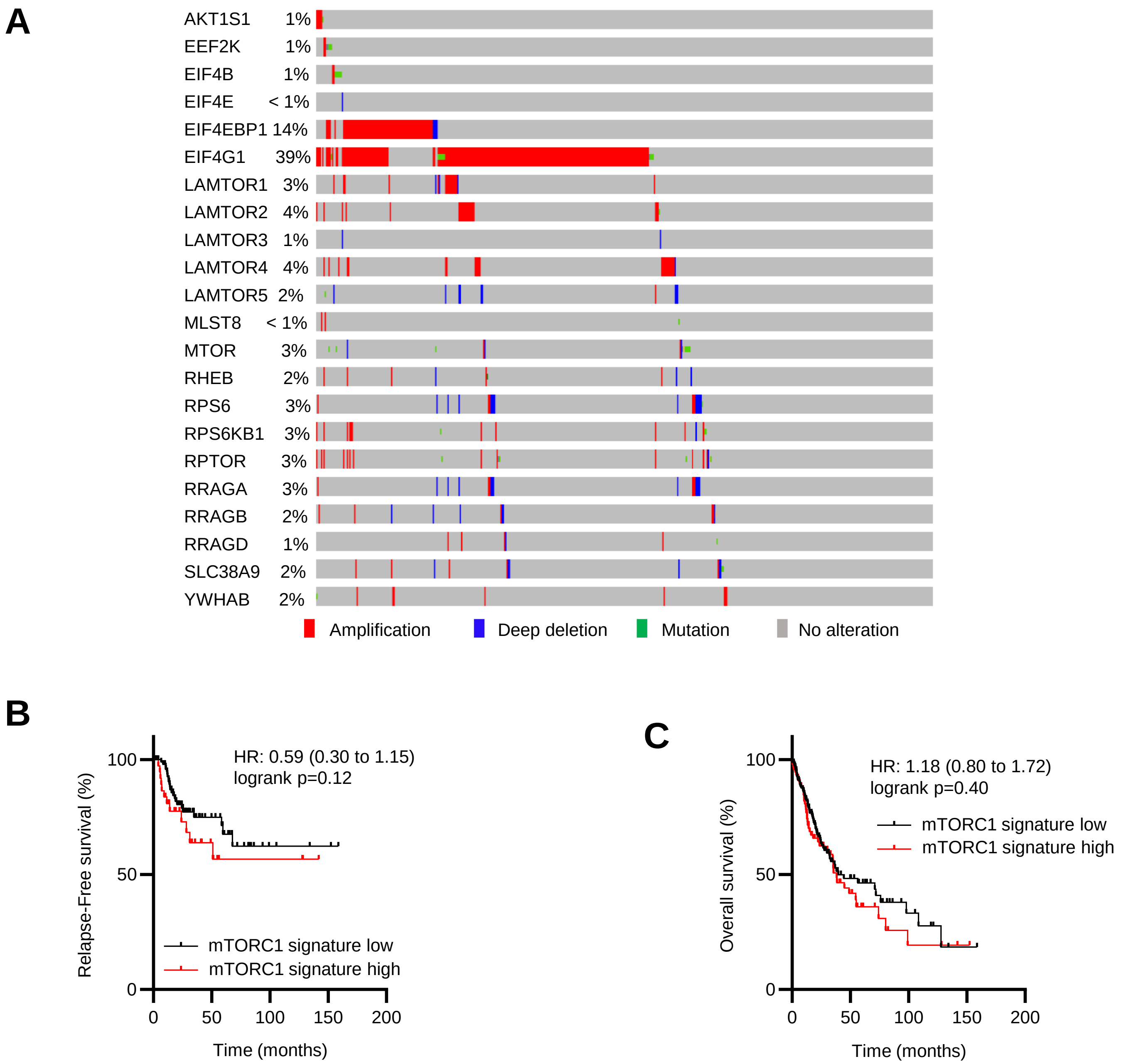

A
AKT1S1 1%
EEF2K 1%
EIF4B 1%
EIF4E < 1%
EIF4EBP1 14%
EIF4G1 39%
LAMTOR1 3%
LAMTOR2 4%
LAMTOR3 1%
LAMTOR4 4%
LAMTOR5 2%
MLST8 < 1%
MTOR 3%
RHEB 2%
RPS6 3%
RPS6KB1 3%
RPTOR 3%
RRAGA 3%
RRAGB 2%
RRAGD 1%
SLC38A9 2%
YWHAB 2%
Amplification
Deep deletion
Mutation
No alteration
B
HR: 0.59 (0.30 to 1.15)
logrank p=0.12
Relapse-Free survival (%)
100
50
0
0
50
100
150
200
Time (months)
mTORC1 signature low
mTORC1 signature high
C
HR: 1.18 (0.80 to 1.72)
logrank p=0.40
mTORC1 signature low
mTORC1 signature high
Overall survival (%)
100
50
0
0
50
100
150
200
Time (months)

D

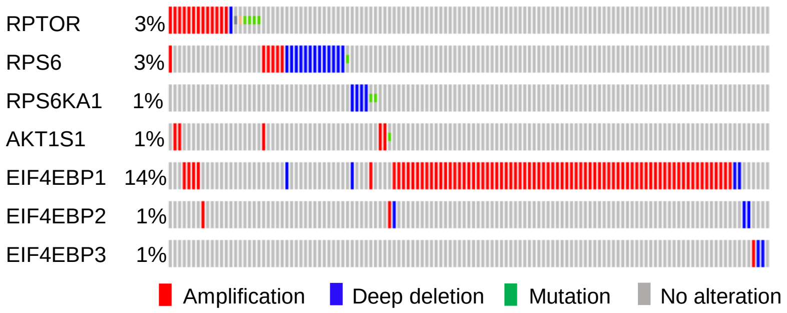

E

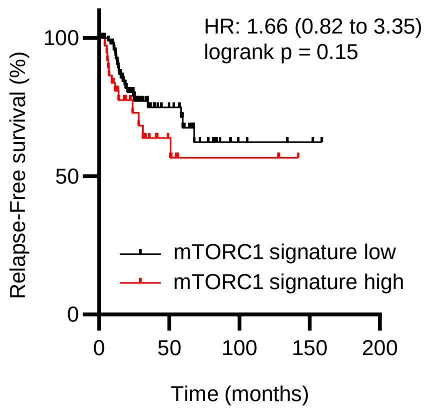

F

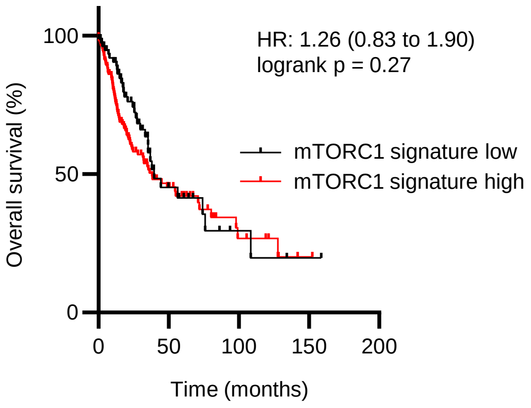

Supplementary Figure 1 related to Figure 1: Genetic alterations in LUSC data analyzing mTORC1 genes. (**A**) Analysis of mTORC1 related genes in the REACTOME_MTORC1_MEDIATED_SIGNALLING gene set in LUSC datasets [**LUSC:** CPTAC (n=80), TCGA Firehose Legacy (n=511)] using cBioPortal. (**B-C**) Kaplan-Meier (KM) of stage II-IV plot displaying the probability of relapse-free survival (**B**) and overall survival (**C**) with association of the mTORC1 related genes illustrated in **A** using lung squamous cell carcinoma RNA-seq datasets (n=141, relapse-free survival; n=249, overall survival) downloaded from kmplot.com. (**D-F**) mTORC1 unique components and downstream genes (mTORC1 signature: *RPTOR*, *RPS6*, *RPS6K*, *A1AKT1S1*, *EIF4EBP1*, *EIF4EBP2*, *EIF4EBP3*) were further analyzed using the same datasets as above. Log-rank (Mantel-Cox) test *p*-values, Hazard ratio (HR) (log-rank) and 95% confidence intervals are shown.

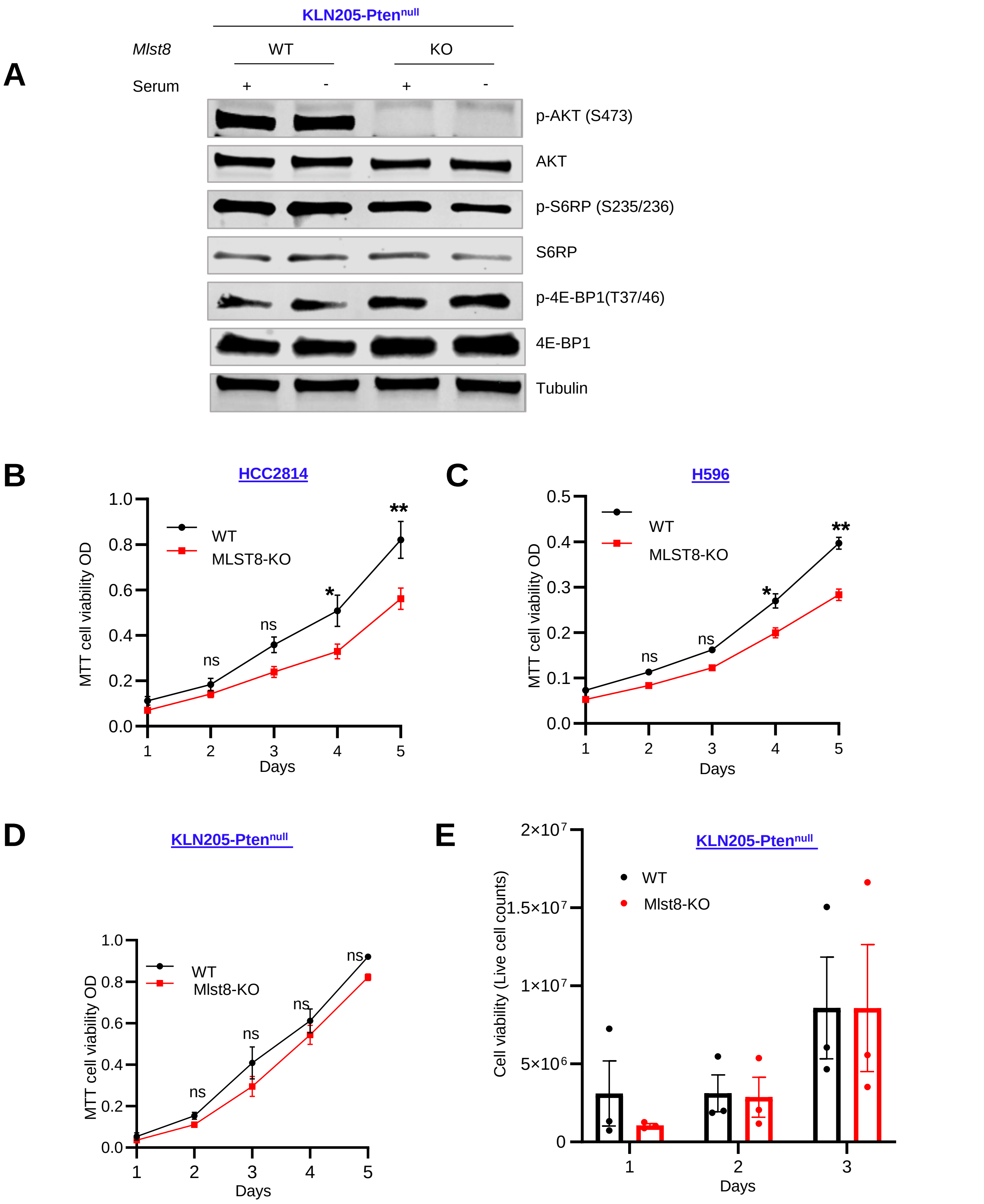

Supplementary Figure 2 related to Figure 2: LUSC invitro cell proliferation. (**A**) KLN205-Pten^{null} WT or Mlst8-KO cells were serum starved overnight, and cell lysates were assessed by western blot analysis. (**B-C**) Cell viability was measured by MTT assay in HCC2814 and H596 WT or MLST8-KO cells (n=3 biological replicates). (**D**) KLN205-Pten^{null} WT or Mlst8-KO cells were cultured for a total of 72 hrs, and dead cells were stained with trypan blue. All data are presented as mean ± SEM from two or three independent experiments. p-values were determined by 2-way ANOVA with Sidak multiple comparisons correction test. *p<0.05, **p<0.01, ns: not statistically significant.

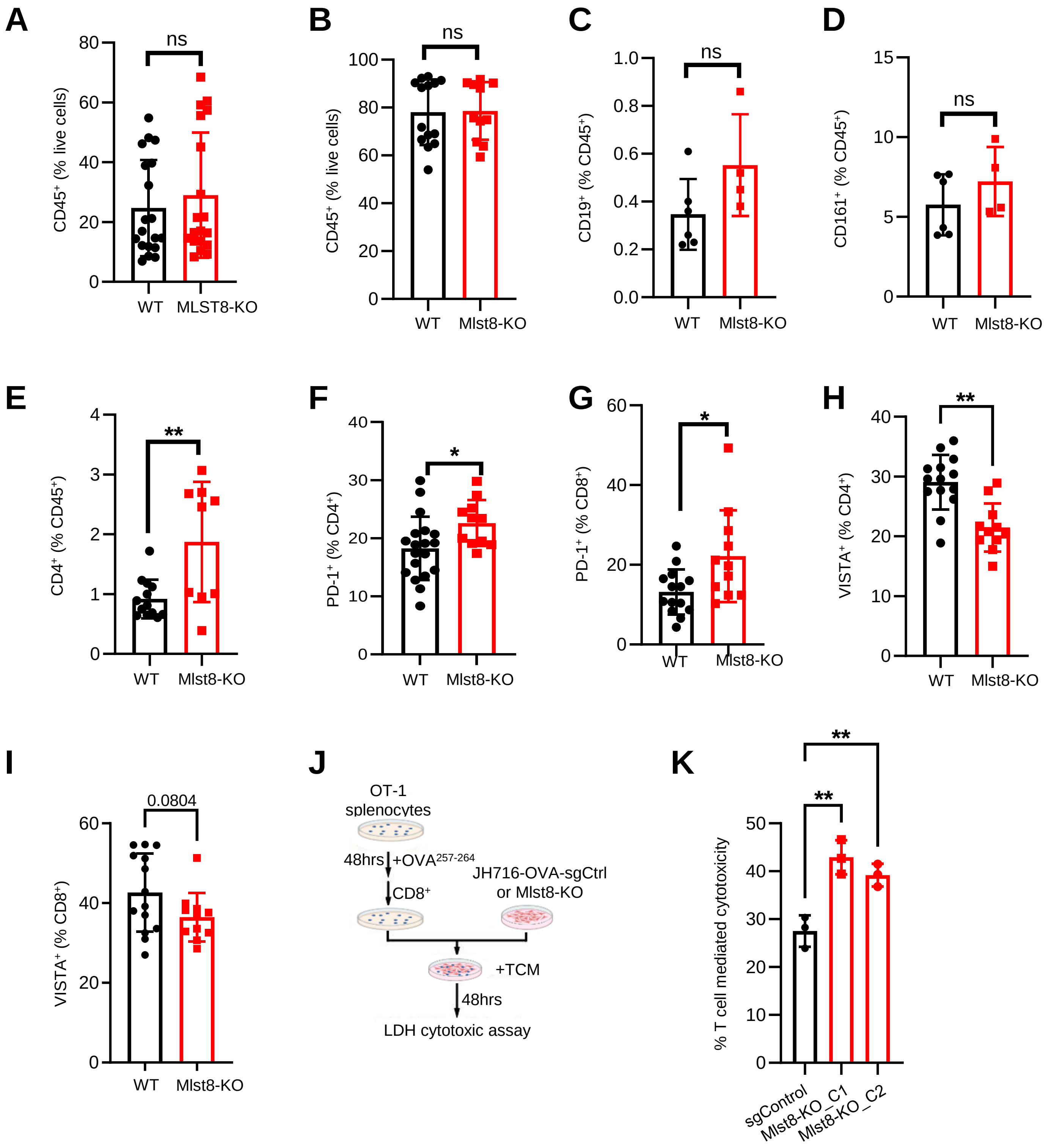
A
CD45+ (% live cells)
ns
WT
MLST8-KO
B
CD45+ (% live cells)
ns
WT
Mlst8-KO
C
CD19+ (% CD45+)
ns
WT
Mlst8-KO
D
CD161+ (% CD45+)
ns
WT
Mlst8-KO
E
CD4+ (% CD45+)
**
WT
Mlst8-KO
F
PD-1+ (% CD4+)
*
WT
Mlst8-KO
G
PD-1+ (% CD8+)
*
WT
Mlst8-KO
H
VISTA+ (% CD4+)
**
WT
Mlst8-KO
I
VISTA+ (% CD8+)
0.0804
WT
Mlst8-KO
J
OT-1 splenocytes
48hrs
+OVA257-264
CD8+
JH716-OVA-sgCtrl or Mlst8-KO
+TCM
48hrs
LDH cytotoxic assay
K
% T cell mediated cytotoxicity
**
**
sgControl
Mlst8-KO_C1
Mlst8-KO_C2

Supplementary Figure 3 related to Figure 3: Effect of MLST8/mTORC2 loss on immune landscape. (A-D) Flow cytometry quantification of CD45$^+$ in H596 tumors (**A**), CD45$^+$ (**B**), B- and NK cells (**C, D**) in KLN205-Pten^{null} WT or Mlst8-KO tumors (n=4-14). (**E**) Flow cytometric analysis of CD4$^+$ (% CD45$^+$) from WT and Mlst8-KO tumors. (**F-I**) Flow cytometric analysis of PD1$^+$ (% CD4$^+$), PD1$^+$ (% CD8$^+$), VISTA$^+$ (% CD4$^+$), VISTA$^+$ (% CD8$^+$) from WT and Mlst8-KO tumors. Each dot represents a mouse. (**J-K**) Cytotoxicity of HJ716-OVA cells was determined. (**J**) Schematic of the co-culture assay is shown. (**K**) JH716-OVA-sgControl vs Mlst8-KO (C1: clone_1 and C2: clone_2) cells were co-cultured with CD8$^+$ T cells in tumor conditioned medium (TCM) (n=3). All data are presented as mean ± SEM from two or three independent experiments. p-values were determined by two-tailed unpaired Student *t* test. *p<0.05, **p<0.01. ns: not statistically significant.

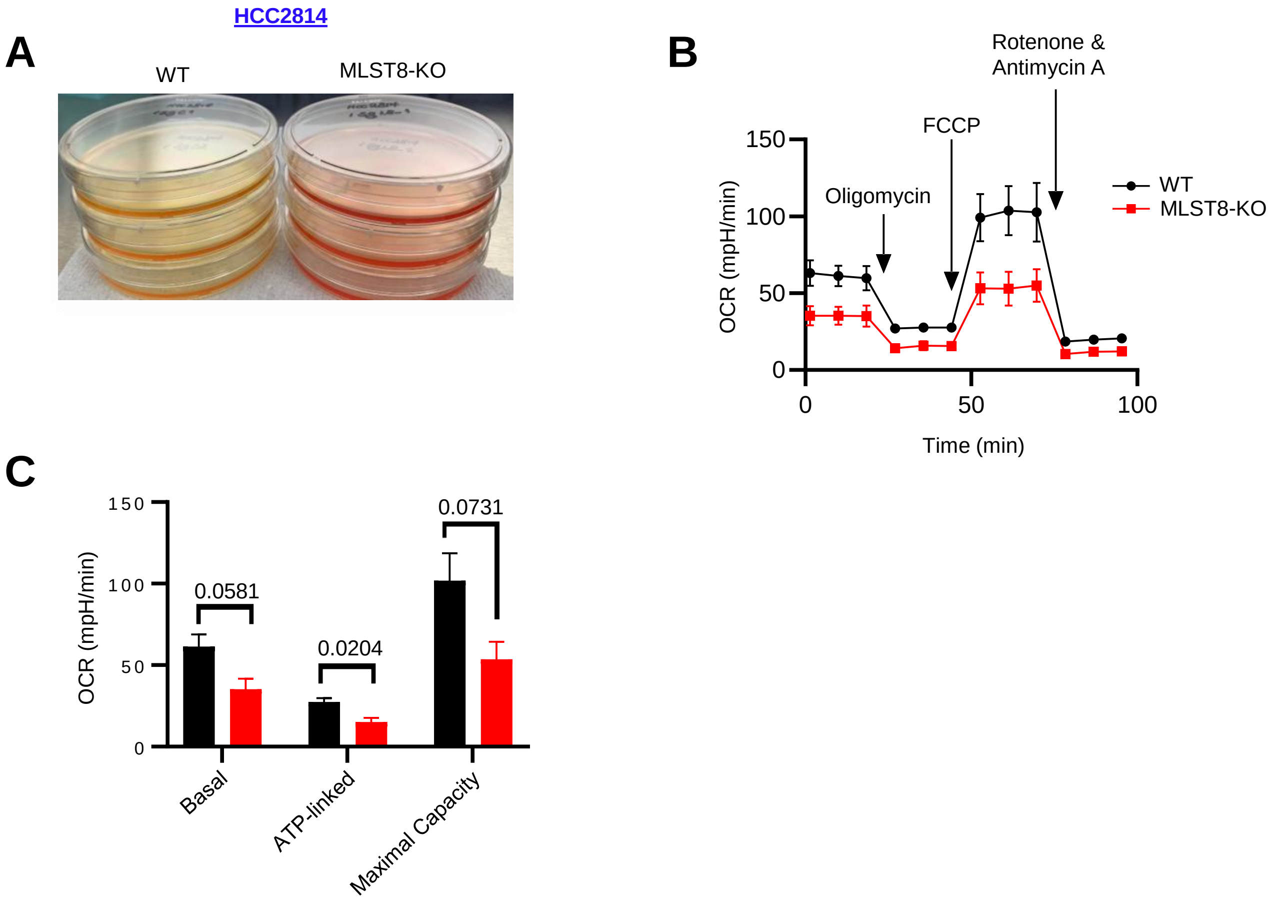

Supplemental Figure 4 related to Figure 4: Loss of function in mTORC2 alters mitochondrial respiration. (**A**) Representative image of media color changes in HCC2814 WT and MLST8-KO cell. (**B**) Oxygen Consumption Rate (OCR) changes in HCC2814 WT and MLST8-KO cells after mLST8 was deleted. (**C**) Bar chart showing mitochondrial respiration function parameters of WT versus MLST8-KO HCC2814 cells analyzed with basal respiration, ATP production and maximal respiration. All data are presented as mean ± SEM from two or three independent experiments. p-values were determined by two-tailed unpaired Student *t* test.

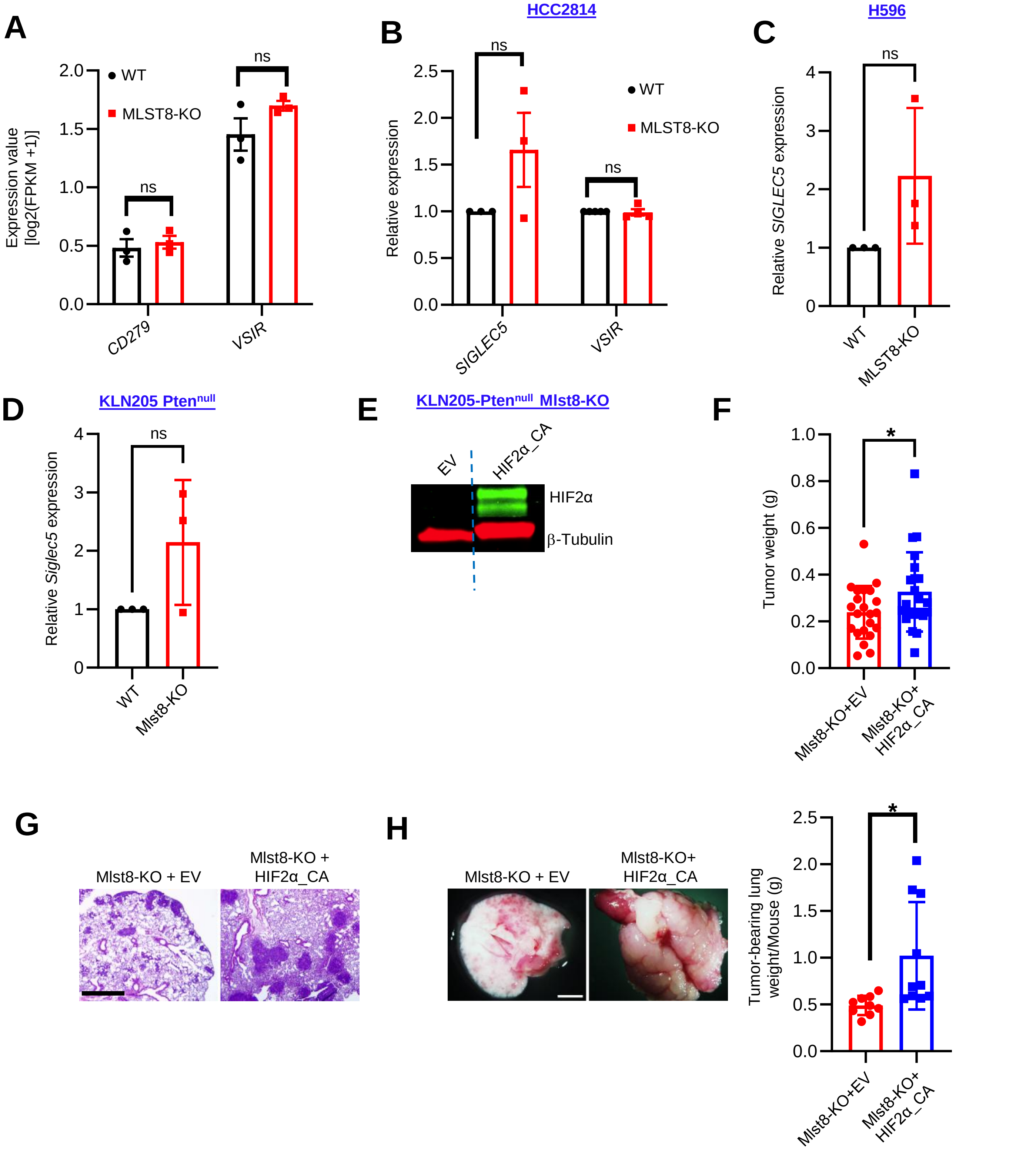

Supplementary Figure 5 related to Figure 5: mTORC2 loss of function does not change other PSGL-1 binding partners but regulates PSGL-1 expression through HIF2α. (**A**) Expression values of *PD-L1* and *VISTA* in MLST8-KO compared to WT from RNA-sequencing. (**B-D**) Expression values of *VISTA* and *SIGLEC-5* by RT-qPCR from HCC2814, H596 and KLN205-Pten^{null} WT or MLST8-KO cells (n=3). (**E**) Western blot of HIF2α from KLN205-Pten^{null} Mlst8-KO cells transfected with Empty Vector (EV) or HIF2α-CA. Blue dotted line denotes spliced gel. (**F**) Subcutaneous tumor weight. (**G-H**) 1x10^6 KLN205-Pten^{null} Mlst8-KO+ EV or Mlst8-KO+ HIF2α-CA cells were injected into DBA/2 mice via tail vein. Representative H&E staining (**G**) and whole lung and weight (**H**) harvested from Mlst8-KO+EV or Mlst8-KO+HIF2α_CA tumor bearing mice after 21 days. Scale bar: 100 µm. Each dot represents a mouse. All data are presented as mean ± SEM from two or three independent experiments. p-values were determined by two-tailed unpaired Student *t* test. *p<0.05, ns: not statistically significant.

KLN205-PTEN^{null} mLST8-KO

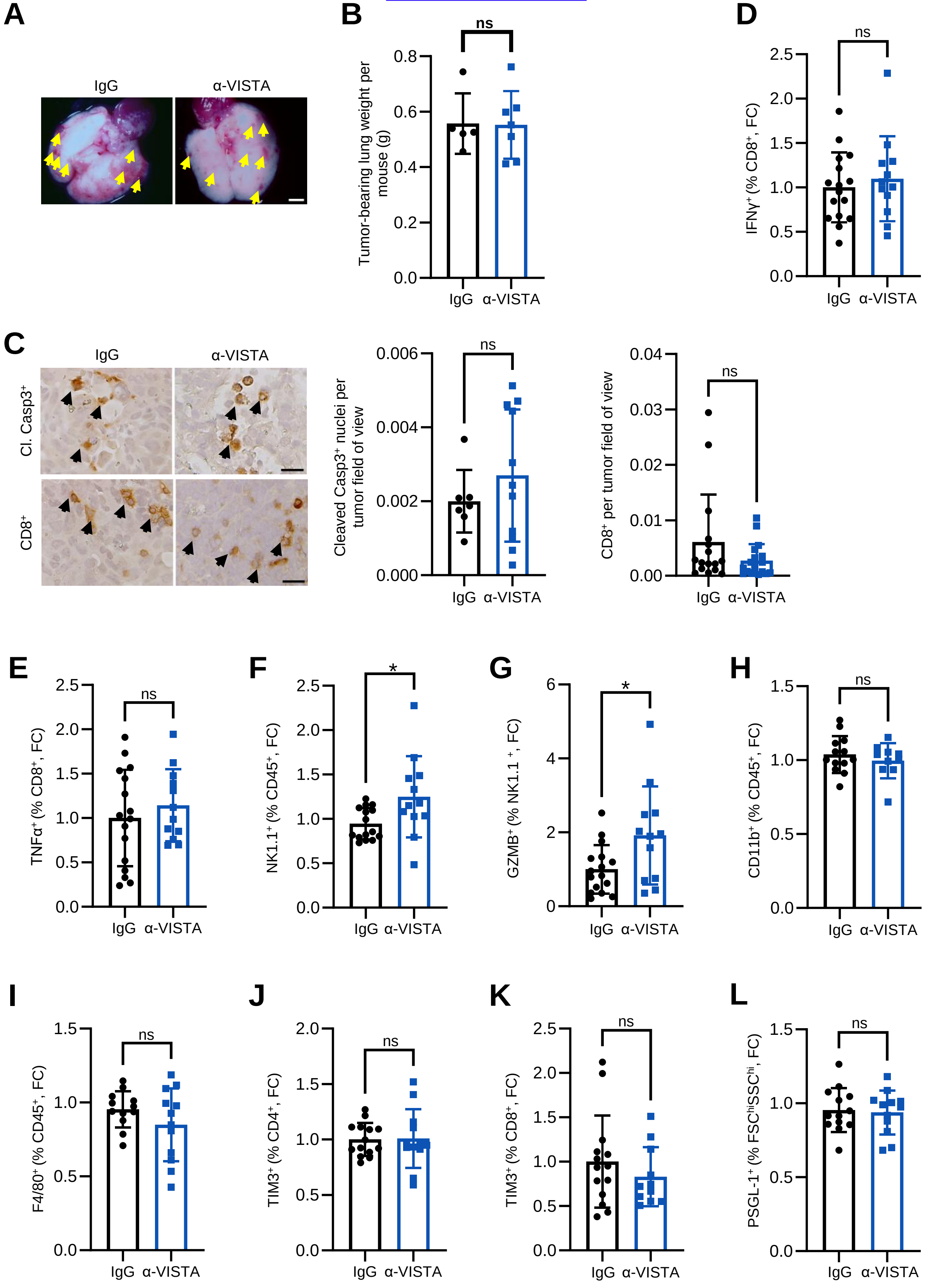

Supplementary Figure 6 related to Figure 6: Anti-VISTA treatment in vivo and in vitro improves T cell cytotoxic killing. (**A**) Representative images of the lungs harvested from IgG control or anti-VISTA treated KLN205-Pten^{null} Mlst8-KO tumor bearing mice after 25 days of tumor cell implantation. Scale bar: 100 μm. (**B**) Lung weight indicating tumor burden from IgG Control and anti-VISTA. Each dot on the quantification represents a mouse. (**C**) Immunohistochemistry identifying Cl. Caspase 3^{+} and CD8^{+} cells on IgG vs anti-VISTA tumor samples. Scale bar: 50 μm. Each dot on the quantification represents Cl. Caspase 3^{+} or CD8^{+} nuclei per tumor field of view. (n=4-6 mice per group). (**D-L**) Flow cytometry was performed on IFNγ^{+}CD8^{+} (**D**), TNF-α^{+}CD8^{+} (**E**), NK1.1^{+}CD45^{+} natural killer cells (**F**), GZMB^{+}NK1.1^{+} (**G**), CD11b^{+} CD45^{+} myeloid cells (**H**), F4/80^{+}CD45^{+} macrophages (**I**), TIM3^{+}CD4^{+} (**J**), TIM3^{+}CD8^{+} (**K**), and PSGL-1^{+} (**L**) from IgG vs anti-VISTA treated mice. Each dot on the quantification represents a mouse. p-values were determined by two-tailed unpaired Student *t* test. *$p<0.05$, ns: not statistically significant.

Supplementary Table 1: Mouse Flow cytometry antibodies used

Antibody	Clone	Dilution	Catalog #	Company
APC-Cy7-CD45	30-F11	1:500	561037	BD Biosciences
PerCP5.5- TCR-β	H57-597	1:250	65-5961	Tonbo/Cytek
CD4 PE/Dazzle-594	GK1.5	1:500	100455	Biolegend
RedFluro710-CD8	53-6.7	1:500	80-0081	Tonbo/Cytek
PE-Cy7-CD25	PC61.5	1:500	60-0251	Tonbo/Cytek
PE-Granzyme B	NGZB	1:20	12-8898-82	eBioscience
PE-CD107a	1D4B	1:300	558661	BD Pharmingen
BV421-VISTA	MIH63	1:500	150212	Biolegend
FITC-PD1	29F.1A12	1:500	135213	Biolegend
PE-CD162/PSGL1	2PH1	1:100	555306	BD Pharmingen
BV605-CD161/NK1.1	PK136	1:100	108739	Biolegend
APC-TNFα	MP6-XT22	1:50	17-7321-82	eBioscience
FITC-CD11b	M1/70	1:250	557396	BD Biosciences
Alexa-Fluor 700-F4/80	BM8	1:250	123129	Biolegend
APC-CD366/TIM3	8B.2C12	1:100	17-5871-80	eBioscience
PE-IgG2a,k	eBR2a	1:20	12-4321-81	eBioscience
APC-IgG1,k	eBRG1	1:50	17-4301-82	eBioscience

Supplementary Table 2: Quantitative RT-PCR Human and Mouse primers used in study

**Human Primers**		
**Genes**	**Forward**	**Reverse**
EPAS1	ATAAGTTCACCCAAAACCCCAT	GGCAGCAGGTAGGACTCAAAT
LDHA	GGATCTCCAACATGGCAGCCTT	AGACGGCTTTCTCCCTCTTGCT
GLUT1	TTGCAGGCTTCTCCAACTGGAC	CAGAACCAGGAGCACAGTGAAG
mLST8	CAGGTGAATGCCTTGGAGGTCA	TTGTTGACGCCGTCGTAGCTGA
SELPLG	ACCCCTGAGTCTACCACTGT	TCCATAGCTGCTGAATCCGT
HK2	GAGTTTGACCTGGATGTGGTTGC	CCTCCATGTAGCAGGCATTGCT
VSIR	GGTGCATCAGAGTGTCCTGCAG	TGCAGGACTAGACGCCAAGATC
SIGLEC5	CACCAACCTCACCTGTCAG	TGCAGGATCTCTAGGGCTATG
ACTB	CATGTACGTTGCTATCCAGGC	CTCCTTAATGTCACGCACGAT
**Mouse Primers**		
**Genes**	**Forward**	**Reverse**
Epas1	GGACAGCAAGACTTTCCTGAGC	GGTAGAACTCATAGGCAGAGCG
Selplg	TTGTGCTGCTGACCATCT	TCCTCAAAATCGTCATCC
Hk2	CAACTCCGGATGGGACAG	CACACGGAAGTTGGTTCCTC
Ldha	GGATGAGCTTGCCCTTGTTGA	GACCAGCTTGGAGTTCGCAGTTA
Siglec5	GCAAGCATAACTTCATCCAAACA	GAGGCATAGTGGAGTTCAGG
Gadph	CCCTCAAGATTGTCAGCAATGC	GTCCTCAGTGTAGCCCAGGAT
